# Simulation and Experimental Study on Doubled-Input Capacitively Coupled Contactless Conductivity Detection of Capillary Electrophoresis

**DOI:** 10.1038/s41598-020-64896-3

**Published:** 2020-05-14

**Authors:** Chunling Wang, Haoyang Xing, Baozhan Zheng, Hongyan Yuan, Dan Xiao

**Affiliations:** 1 College of Chemical Engineering, Sichuan University, Chengdu, 610064 People’s Republic of China; 2 College of Physical Science and Technology, Sichuan University, Chengdu, 610065 People’s Republic of China; 3 College of Chemistry, Sichuan University, Chengdu, 610065 People’s Republic of China

**Keywords:** Chemistry, Analytical chemistry

## Abstract

In this contribution, we optimize the structure of double-input capacitively coupled contactless conductivity detector (DIC^4^D) that proposed before by our group and successfully applied it in the capillary electrophoresis of inorganic ion analysis. Furthermore, we present the detail theoretical analysis and simulation to exploring the working mechanism of DIC^4^D. Compared with C^4^D, under identical experimental conditions and by using the same current-to-voltage converter, both the theoretical and experimental results suggest that the effectiveness and feasibility of DIC^4^D. The improved DIC^4^D diminished the baseline drift effects in C^4^D, provides lower noise, higher sensitivity and notably stable baseline. The LODs of DIC^4^D are 1.0 μM for K^+^ and 1.5 μM for Li^+^ (S/N = 3). DIC^4^D provides a better linear relationship (R = 0.997 and 0.998 for K^+^ and Li^+^, respectively) with the range of 2.0 μM ~ 2.5 mM.

## Introduction

As a particular type of conductivity detector, the capacitively coupled contactless conductivity detector (C^4^D) has the advantage of being free from contamination because it is not in direct contact with the measured solution. Thus, the applications of C^4^D for electroseparations have received considerable attention in fields of medicine, environment, food testing and biology *et al*.^1–5^

Zemann *et al*.^6^ and Fracassi da Silva *et al*.^7^ proposed the use of C^4^D in capillary zone electrophoresis and proved the advantages of C^4^D. Since then, much effort has been made to improve the performance of the contactless conductivity detectors. Do Lago *et al*.^8^ improved the hardware and optimized the operational parameters. Tanyanyiwa *et al*.^9,10^ applied a relatively high-input alternating current (AC) voltage (250 ~ 450 Vpp) to increase the sensitivity of C^4^D. Hauser *et al*.^11^ tested C^4^D using a capillary made from Polyetheretherketone and obtained similar results with a fused silica capillary or glass capillary. Kang *et al*.^12,13^ adopted an inductance coupling device to measure the conductivity of C^4^D and proposed a new C^4^D, which is compensated with the electrode impedance by adding a series inductance from a piezoelectric quartz crystal to decrease the coupling impedance and increase sensitivity. Tuma *et al*.^14^ designed a new C^4^D device with an easily exchangeable capillary for capillary electrophoresis. Fercher *et al*.^15^ proposed an end-to-end differential capacitively coupled contactless conductivity measurement, which can obtain high peak-to-baseline ratio (P/B) and signal-to-noise ratio (S/N). Mendonca *et al*.^16^ improved the electronic circuit of C^4^D using low noise circuitry and a high-resolution analog to digital converter to get lower limits of detection (LODs). Jaanus *et al*.^17^ and Stojkovic *et al*.^18^ proposed two electrode pairs C^4^D detector, which are arranged in two different capillary channels. This improvement provided an effective compensation which can suppress of the influence of the parasitic stray capacitance and decrease the baseline conductivity of the separation buffer.

Other improvements include miniaturization, integration, shielding and decreasing the stray capacitance^19,20^. Because it is easily miniaturized, integrated and portable and can be applied on substrates such as glass or polymer materials, some research groups have begun the application of C^4^D in microchips and improved microchip C^4^D in the aspects of structure, detection circuits and electrode process^21–25^.

A new C^4^D structure named DIC^4^D proposed before by Zheng in our group, which can improve the sensitivity via the conductive difference of fluid between two electrode pairs. However, Zheng did not provide sufficiently theoretical explain and the application of DIC^4^D. Zheng also showed that the signal of DIC^4^D was affected by several factors, including the input signal voltage, phase difference of two input signals, distance between two electrodes and waveform of the input alternating signal *et al*. These effects can result in calculation errors, which is unfavorable to quantitative analysis.

**Figure 1:**
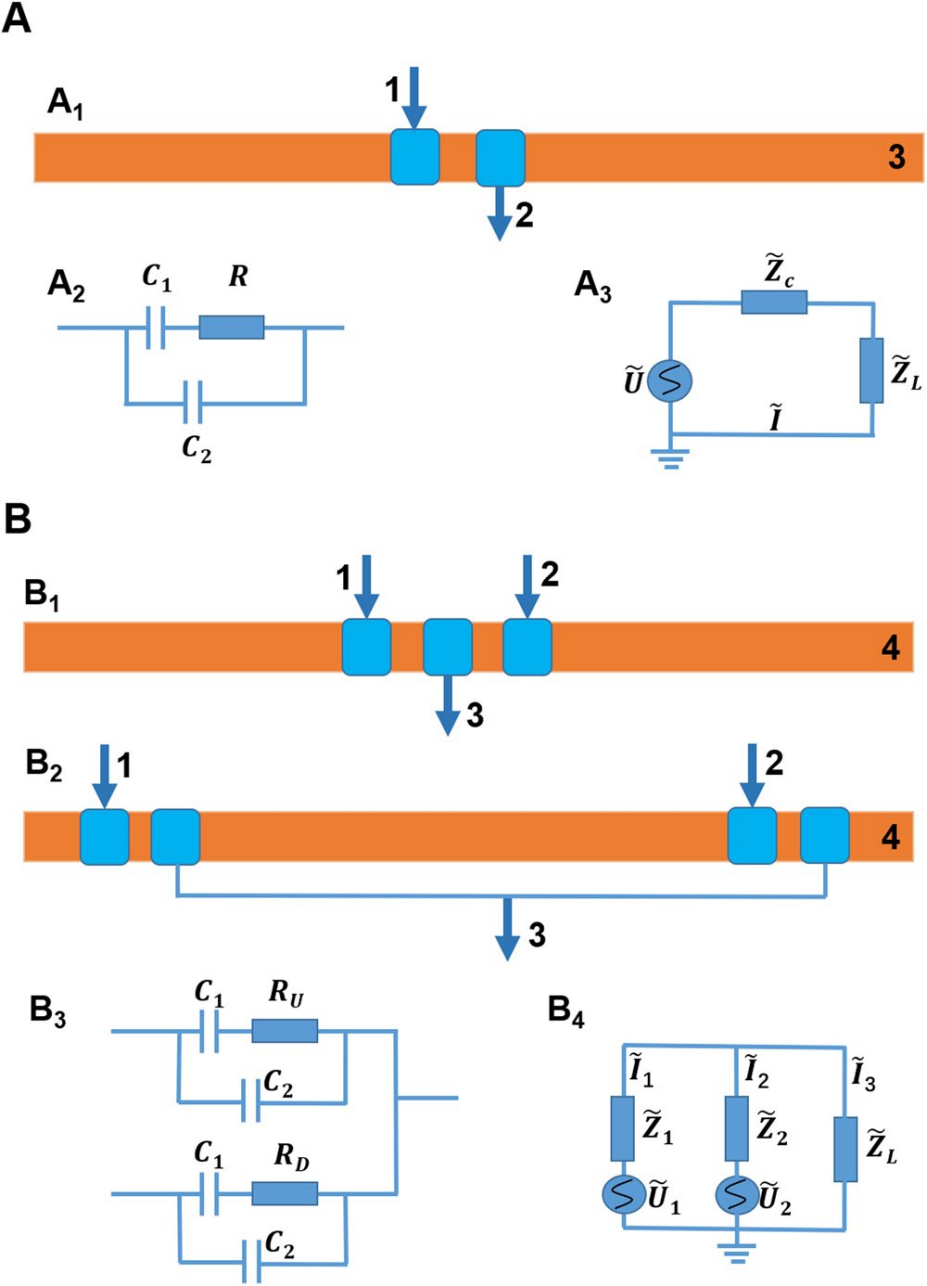
Schematic illustration of the structure, equivalent circuit and entire circuit model of C^4^D (A) and DIC^4^D (B). Fig. 1A_1_ is the structure of C^4^D. 1. Input electrode. 2. Output electrode. 3. Separation capillary. Fig. 1A_2_ and A_3_ are the equivalent circuit and entire circuit model of C^4^D, respectively. Fig. 1B_1_ and B_2_ are the structure of three electrodes and four electrodes DIC^4^D, respectively. 1 and 2. Input electrodes. 3. Output electrode. 4. Separation capillary. Fig. 1B_3_ and B_4_ are the equivalent circuit and entire circuit model of a generic DIC^4^D, respectively.

Regarding to these problems, in this study, the equivalent circuit of DIC^4^D was simulated by using the tool MultiSim, and the influence factors of the performance were analyzed considering the entire detection circuit (including the AC voltage source, impedance cells, and collection circuit of signals). In our previous work, the structure of DIC^4^D proposed by Zheng^26^ is three electrodes DIC^4^D (Fig. 1B_1_). Here, an improvement of four electrodes DIC^4^D was proposed: replacing two successive electrode pairs on the capillary tube with two separated electrode pairs (Fig. 1B_2_). The three electrodes DIC^4^D and four electrodes DIC^4^D both have the same equivalent circuits and entire circuits. (Fig. 1B_3_,1B_4_). The mechanism of the DIC^4^D was explained according to the simulation results. The improvement of the DIC^4^D structure overcomes the limitation of the original DIC^4^D and maintains the sensitivity. If not specified, the following DIC^4^D refer to the improved DIC^4^D (four electrodes DIC^4^D). Furthermore, the Peak-to-Baseline Ratio (P/N), sensitivity, and Signal-to-Noise Ratio (S/N) of DIC^4^D were investigated by a series of experiments to detect the inorganic ions K^+^ and Li^+^.

## Results and Discussion

### Theoretical section

Because the C^4^D uses alternating current (usually sine-wave) voltage and depends on capacitive coupling, the analysis here is in the complex form. In the complex form of the AC circuit, the voltage is $\tilde{U}=U{e}^{j(\omega t+{\varphi }_{U})}$. Replacing $U{e}^{j\omega t}$ with ${\tilde{U}}_{0}$, according to Euler’s law, $\tilde{U}={\tilde{U}}_{0}{e}^{j{\varphi }_{U}}={\tilde{U}}_{0}(cos{\varphi }_{U}+jsin{\varphi }_{U})$. The impedance is $\tilde{Z}=Z{e}^{j{\varphi }_{Z}}$, and the current is $\tilde{I}=\frac{\tilde{U}}{\tilde{Z}}=\frac{{\tilde{U}}_{0}}{Z}{e}^{j({\varphi }_{U}-{\varphi }_{Z})}$.

### Classical C^4^D

The classical C^4^D impedance cell consists of a capillary tube and a pair of detection electrodes. The AC signal is transferred by the coupling of capacitance and conductivity between two electrodes. The structure of C^4^D is demonstrated in Fig. 1A_1_; the equivalent circuit^7^ and entire circuit model are shown in Fig. 1A_2_,A_3_.

In Fig. 1A_2_, ${C}_{1}$ is the capacitance formed with a glass tube wall, and ${C}_{2}$ is the stray capacity formed with a glass wall and air between two electrodes. *R* is the resistance formed with the electrical conductivity of the test medium in the capillary tube.

For C^4^D, the total complex impedance ${\tilde{Z}}_{C}$ of the circuit is

1 $${\tilde{Z}}_{C}=\frac{1+j\omega {C}_{1}R}{j\omega {C}_{1}+j\omega {C}_{2}+{\omega }^{2}{C}_{1}{C}_{2}R}$$

The entire C^4^D circuit must include the AC signal source and electronic detection circuit, which can be represented with the circuit model in Fig. 1A_3_. Here, $\tilde{U}$ is the AC voltage source, and ${\tilde{Z}}_{L}$ is the load formed by the detection circuit. The output signal is

2 $${\tilde{S}}_{C4D}=\tilde{I}{\tilde{Z}}_{L}=\frac{\tilde{U}}{{\tilde{Z}}_{C}+{\tilde{Z}}_{L}}{\tilde{Z}}_{L}$$

The amplitude of the output signal is

3 $${S}_{C4D}=|\tilde{I}{\tilde{Z}}_{L}|=|\frac{\tilde{U}}{{\tilde{Z}}_{C}+{\tilde{Z}}_{L}}|{Z}_{L}=\frac{U}{|{\tilde{Z}}_{C}+{\tilde{Z}}_{L}|}{Z}_{L}$$

### DIC^4^D

Compared to C^4^D, DIC^4^D has two signal inputs. As demonstrated in Fig. 1B_1_,B_2_, 1 and 2 in Fig. 1B are input electrodes. 3 in Fig. 1B is output electrode. The length, width, shape, and material of the three electrodes were identical. The gaps of every two electrodes had identical widths. Correspondingly, the equal circuit of DIC^4^D and the entire DIC^4^D equivalent circuit, which include the signal source and detection circuit, are shown in Fig. 1B_2_,B_3_. The three electrodes DIC^4^D and four electrodes DIC^4^D both have the same equivalent circuits and entire circuits.

The circuit of DIC^4^D, which includes the AC signal source and detection circuit, is a complex net circuit. ${\tilde{Z}}_{1}$ and ${\tilde{Z}}_{2}$ are the impedances of the upper and lower branches, which consist of the branch capacitance ${C}_{1}$ from the glass tube wall, stray capacity ${C}_{2}$ and conductive medium resistances *R*_*U*_ and *R*_*D*_ in Fig. 1B_2_. ${\tilde{Z}}_{L}$ is also the load formed by the detection circuits, ${\tilde{I}}_{1}$ and ${\tilde{I}}_{2}$ are the currents of the upper and lower branches, respectively, whose directions were assigned to flow into the junction point ${\tilde{I}}_{3}$ is the current that flows out from the junction point, which is also the current passing through detection load ${\tilde{Z}}_{L}$. Obviously, the signal is the product of ${\tilde{I}}_{3}$ and ${\tilde{Z}}_{L}$.

According to Kirchhoff’s Junction Rule, the signal ${\tilde{I}}_{3}{\tilde{Z}}_{L}$ is

4 $${S}_{DIC4D}=|{\tilde{I}}_{3}{\tilde{Z}}_{L}|=\frac{|{\tilde{U}}_{1}{\tilde{Z}}_{2}{\tilde{Z}}_{L}-{\tilde{U}}_{2}{\tilde{Z}}_{1}{\tilde{Z}}_{L}|}{|{\tilde{Z}}_{1}{\tilde{Z}}_{2}+{\tilde{Z}}_{1}{\tilde{Z}}_{L}+{\tilde{Z}}_{2}{\tilde{Z}}_{L}|}$$

Selecting two identical-magnitude AC sources with inversed phases and using *U* to represent their amplitude, the signal amplitude can be written as

5 $${S}_{DIC4D}=\frac{U{Z}_{L}|{\tilde{Z}}_{2}{e}^{j(-{\varphi }_{U})}+{\tilde{Z}}_{1}{e}^{j(\pi -{\varphi }_{U})}|}{|{\tilde{Z}}_{1}{\tilde{Z}}_{2}+{\tilde{Z}}_{1}{\tilde{Z}}_{L}+{\tilde{Z}}_{2}{\tilde{Z}}_{L}|}=\frac{U{Z}_{L}|{\tilde{Z}}_{2}-{\tilde{Z}}_{1}|}{|{\tilde{Z}}_{1}{\tilde{Z}}_{2}+{\tilde{Z}}_{1}{\tilde{Z}}_{L}+{\tilde{Z}}_{2}{\tilde{Z}}_{L}|}$$

### Peak-to-Baseline Ratio (P/N)

For C^4^D, when there is only background electrolyte in the capillary, the state can be considered a background. The resistor is *R*_*B*_, and the complex impedance of the tube that contains background electrolyte is

$${\tilde{Z}}_{B}=\frac{1+j\omega {C}_{1}{R}_{B}}{j\omega {C}_{1}+j\omega {C}_{2}+{\omega }^{2}{C}_{1}{C}_{2}{R}_{B}}$$

When analytes pass through the electrodes, the resistor is *R*_*A*_, and the reactance of the tube that contains analyte ions is

$${\tilde{Z}}_{B}=\frac{1+j\omega {C}_{1}{R}_{A}}{j\omega {C}_{1}+j\omega {C}_{2}+{\omega }^{2}{C}_{1}{C}_{2}{R}_{A}}$$

Correspondingly, ${\tilde{Z}}_{C}$ in Fig. 1A_3_ changes from ${\tilde{Z}}_{B}$ to ${\tilde{Z}}_{A}$. The coupled signal of the front state can be considered the background; the difference in signal between the front state and the latter state is the peak of the signal. Thus, the P/N of C^4^D (P/N_C_^4^_D_) can be described as

6 $${\rm{P}}/{{\rm{N}}}_{C4D}=\frac{\frac{U}{|{\tilde{Z}}_{A}+{\tilde{Z}}_{L}|}{Z}_{L}}{\frac{U}{|{\tilde{Z}}_{B}+{\tilde{Z}}_{L}|}{Z}_{L}}=\frac{|{\tilde{Z}}_{B}+{\tilde{Z}}_{L}|}{|{\tilde{Z}}_{A}+{\tilde{Z}}_{L}|}$$

For DIC^4^D, when there is only background electrolyte in the capillary, both conductivities in the two branches are identical, and ${\tilde{Z}}_{1}$ and ${\tilde{Z}}_{2}$ are equal to ${\tilde{Z}}_{B}$. Thus, according to Eq. (5),

$$|{\tilde{I}}_{3B}{\tilde{Z}}_{L}|=\frac{U{Z}_{L}|{\tilde{Z}}_{B}-{\tilde{Z}}_{B}|}{|{\tilde{Z}}_{B}{\tilde{Z}}_{B}+{\tilde{Z}}_{B}{\tilde{Z}}_{L}+{\tilde{Z}}_{B}{\tilde{Z}}_{L}|}=0$$

In this case, the minimum value of the signal is 0.

When analytes pass through one of the electrode pairs,

$$|{\tilde{I}}_{3A}{\tilde{Z}}_{L}|=\frac{U{Z}_{L}|{\tilde{Z}}_{B}-{\tilde{Z}}_{A}|}{|{\tilde{Z}}_{A}{\tilde{Z}}_{B}+{\tilde{Z}}_{A}{\tilde{Z}}_{L}+{\tilde{Z}}_{B}{\tilde{Z}}_{L}|} > 0$$

If ${\tilde{Z}}_{B}$ is not equal to ${\tilde{Z}}_{A}$, the above equation is not 0. Hence,

7 $${\rm{P}}/{{\rm{N}}}_{DIC4D}=\frac{|{\tilde{I}}_{3A}{\tilde{Z}}_{L}|}{|{\tilde{I}}_{3B}{\tilde{Z}}_{L}|}=\infty $$

Here, we can see that P/N_DIC_^4^_D_ is theoretically infinitely large. In fact, the two phase-inversed signals cannot totally offset each other, and there is also a noise current because of the induction from the outside magnetic wave and other electronic noise^27^. Because of these factors, the measured baseline in the DIC^4^D is on the 0.09 mV level. Compared to the *I*_*B*_ in C^4^D, it is significantly smaller.

### Signal-to-Noise Ratio (S/N)

Because the change caused by analytes is only the difference of output signals in two cases, the effective signal of C^4^D should be the signal change caused by the difference between ${\tilde{Z}}_{A}$ and ${\tilde{Z}}_{B}$. Considering a noise current *I*_*N*_, the S/N is

8 $${\rm{S}}/{{\rm{N}}}_{C4D}=\frac{\frac{U}{|{\tilde{Z}}_{A}+{\tilde{Z}}_{L}|}{Z}_{L}-\frac{U}{|{\tilde{Z}}_{B}+{\tilde{Z}}_{L}|}{Z}_{L}}{{I}_{N}{Z}_{L}}=\frac{U(\frac{|{\tilde{Z}}_{B}+{\tilde{Z}}_{L}|-|{\tilde{Z}}_{A}+{\tilde{Z}}_{L}|}{|{\tilde{Z}}_{A}{\tilde{Z}}_{B}+{\tilde{Z}}_{A}{\tilde{Z}}_{L}+{\tilde{Z}}_{B}{\tilde{Z}}_{L}+{{\tilde{Z}}_{L}}^{2}|})}{{I}_{N}}$$

For DIC^4^D, the baseline is 0 in the ideal condition, so the effective signal is almost the output signal.

$${\rm{S}}/{{\rm{N}}}_{DIC4D}=\frac{|{\tilde{I}}_{3}{\tilde{Z}}_{L}|}{{I}_{N}{Z}_{L}}=\frac{\frac{U{Z}_{L}|{\tilde{Z}}_{2}-{\tilde{Z}}_{1}|}{|{\tilde{Z}}_{1}{\tilde{Z}}_{2}+{\tilde{Z}}_{1}{\tilde{Z}}_{L}+{\tilde{Z}}_{2}{\tilde{Z}}_{L}|}}{{I}_{N}{Z}_{L}}$$

We replace ${\tilde{Z}}_{1}$ and ${\tilde{Z}}_{2}$ with ${\tilde{Z}}_{B}$ and ${\tilde{Z}}_{A}$, respectively.

9 $${\rm{S}}/{{\rm{N}}}_{DIC4D}=\frac{\frac{U|{\tilde{Z}}_{B}-{\tilde{Z}}_{A}|}{|{\tilde{Z}}_{A}{\tilde{Z}}_{B}+{\tilde{Z}}_{A}{\tilde{Z}}_{L}+{\tilde{Z}}_{B}{\tilde{Z}}_{L}|}}{{I}_{ND}}$$

Comparing the absolute value of the numerator in Eq. (8) to Eq. (9), because ${\tilde{Z}}_{L}$ is the main resistance component, ${\tilde{\,Z}}_{L}^{2}$ is almost a real number. Thus, with the realistic parameters of the C^4^D experiment, we have

10 $$\frac{|{\tilde{Z}}_{B}+{\tilde{Z}}_{L}|-|{\tilde{Z}}_{A}+{\tilde{Z}}_{L}|}{|{\tilde{Z}}_{A}{\tilde{Z}}_{B}+{\tilde{Z}}_{A}{\tilde{Z}}_{L}+{\tilde{Z}}_{B}{\tilde{Z}}_{L}+{{\tilde{Z}}_{L}}^{2}|} < \frac{|{\tilde{Z}}_{B}-{\tilde{Z}}_{A}|}{|{\tilde{Z}}_{A}{\tilde{Z}}_{B}+{\tilde{Z}}_{A}{\tilde{Z}}_{L}+{\tilde{Z}}_{B}{\tilde{Z}}_{L}+{{\tilde{Z}}_{L}}^{2}|} < \frac{|{\tilde{Z}}_{B}-{\tilde{Z}}_{A}|}{|{\tilde{Z}}_{A}{\tilde{Z}}_{B}+{\tilde{Z}}_{A}{\tilde{Z}}_{L}+{\tilde{Z}}_{B}{\tilde{Z}}_{L}|}$$

This formula shows that the DIC^4^D has a larger effective signal than the C^4^D in some test conditions. The comparison of the front two items suggests that the phases of the complex recantations is a useful contribution to the signal magnitude because the magnitude of the resultant vector must be larger than the difference in absolute value of the two vectors. In the classical C^4^D, only the magnitude is useful, and the phase is disregarded. The comparison of the latter two items shows that the DIC^4^D signal has a larger amplitude than the C^4^D signal.

In addition to the larger signal intensity, an advantage of DIC^4^D is the suppression of some common mode noise, which can be offset because the noise occurs on both detectors. Note that the offset common mode noise here is only caused by the factors that simultaneously affect the magnitudes of two currents ${\tilde{I}}_{1}$ and ${\tilde{I}}_{2}$. Although DIC^4^D has one more branch, the other noise does not obviously increase because of the random property of noise. The noise of DIC^4^D is less than or at least at the same level as that of C^4^D. Thus, ${\rm{S}}/{{\rm{N}}}_{{\rm{DIC}}4{\rm{D}}}$>${\rm{S}}/{{\rm{N}}}_{{\rm{C}}4{\rm{D}}}$ according to the above derivation.

### Sensitivity

The detection sensitivity is the amplitude change rate with respect to the change in analyte concentration:^28^

$${\rm{S}}=\frac{\Delta S}{\Delta C}$$

For C^4^D,

11 $${{\rm{S}}}_{C4D}=\frac{\frac{U}{|{\tilde{Z}}_{A}+{\tilde{Z}}_{L}|}{Z}_{L}-\frac{U}{|{\tilde{Z}}_{B}+{\tilde{Z}}_{L}|}{Z}_{L}}{\Delta C}$$

For DIC^4^D,

12 $${{\rm{S}}}_{DIC4D}=\frac{\frac{U|{\tilde{Z}}_{B}-{\tilde{Z}}_{A}|}{|{\tilde{Z}}_{A}{\tilde{Z}}_{B}+{\tilde{Z}}_{A}{\tilde{Z}}_{L}+{\tilde{Z}}_{B}{\tilde{Z}}_{L}|}}{\Delta C}$$

Compared to C^4^D, because of the larger signal intensity according to Formula (10), DIC^4^D has higher sensitivity.

Therefore, DIC^4^D is an improved structure based on the idea of differential calculation; the baseline signal from the surrounding noise can be notably small or even cancelled to 0 when the two signals have opposite phases in an ideal condition without electromagnetic interference. The S/N and sensitivity are improved because of the new circuit structure.

### Simulation results

**Figure 2:**
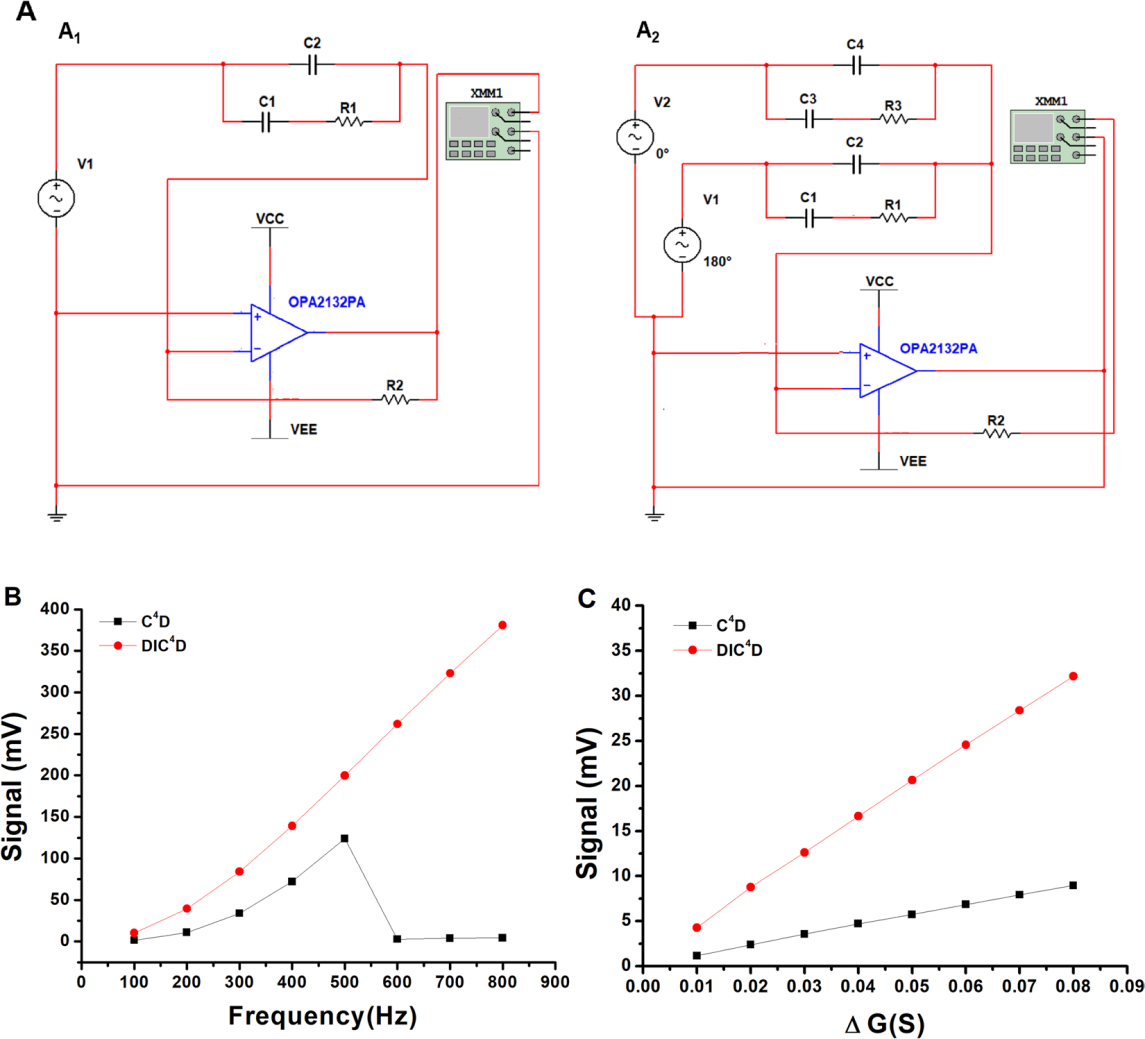
Schematic diagram of the simulation circuit and results for C^4^D and DIC^4^D. Fig. 2A_1_ and A_2_ are the simulation circuit for C^4^D and DIC^4^D, respectively. Fig. 2B is the simulated coupled signals of C^4^D and DIC^4^D at different frequencies. Fig. 2C is the simulated signals of C^4^D and DIC^4^D at different conductivities corresponding to different analyte concentrations at 200 kHz work frequency.

The above analysis of the equivalent circuits of C^4^D and DIC^4^D is only qualitative. So, we performed a simulation implemented to quantitatively investigate the response of the circuit model at the approximately real circuit parameters. The simulation results are shown in Fig. 2B, where the coupled signal changed with the resistance R1 at different frequencies. Figure 2B shows that the DIC^4^D had obviously larger signals than C^4^D at all investigated frequencies for the 10% conductance change of R1. When the frequency was less than 500 kHz, both C^4^D and DIC^4^D increased the signals with increasing work frequency. After 500 kHz, the DIC^4^D signal continued to increase, but the C^4^D signal sharply decreased probably because of the saturation of the transimpedance circuit.

The response signal to different conductivities depending on the analyte concentrations is demonstrated in Fig. 2C. Both C^4^D and DIC^4^D have good linearity in the investigated range of conductivities (the correlation coefficients are larger than 0.999). Compared to C^4^D, the sharper slope rate shows that DIC^4^D also has better sensitivity to the change in R1.

The simulation in this section illustrates that DIC^4^D has a relatively higher signal than C^4^D, which also indicates better S/N and sensitivity. However, the above derivation and simulation are based on the pre-requirement of the symmetry of two branches, and the analyte simultaneously appears in two electrode pairs. In fact, the symmetry of two branches is notably difficult to ensure because of the fabrication. Thus, the amplitudes and phases of the AC source must be adjusted to maintain a zero baseline and achieve a higher P/N. In Zheng’s experiments, the suitable phase difference is 170°^26^. Because there is a short distance between two electrode pairs, if the analytes distribute in a relatively wide range and cover two pairs of electrodes, the signal is probably canceled by the same change in *R*_*U*_ and *R*_*D*_ or strongly interfered with each other, leading to a distortion in the waveform of the signal peak. This phenomenon was observed and described in the work of Zheng^26^. Hence, an improvement was proposed in this work to eliminate the effect of the transition time. We put the electrode pairs near two ends of the capillary tube (Fig. 1B_2_). Compared to the original DIC^4^D (Fig. 1B_1_), the separation in space for two pairs of electrodes avoids the signal interference between two pairs of electrodes in the original structure.

### Sample analysis

**Figure 3:**
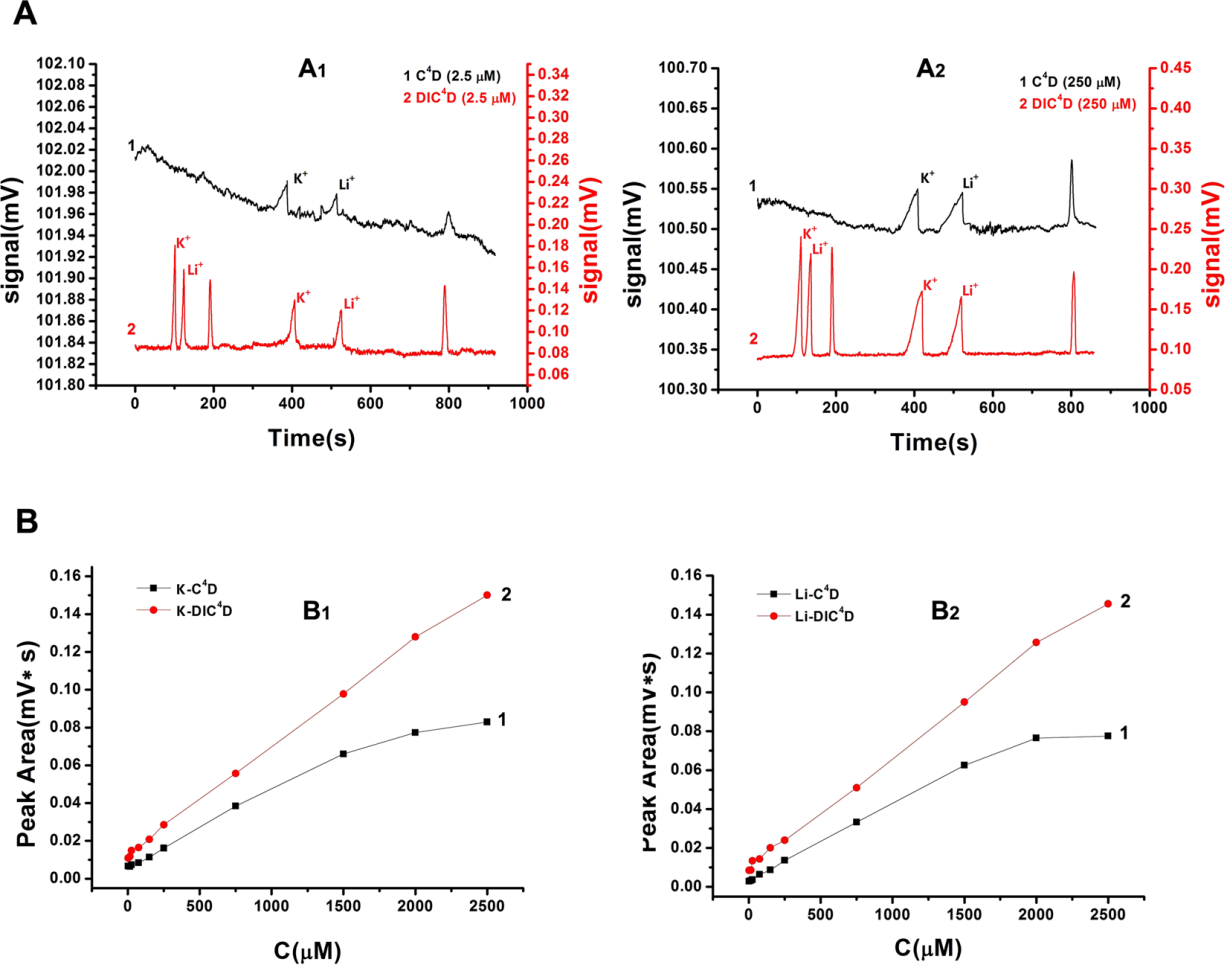
Electrophoresis experimental results of K^+^ and Li^+^ with C^4^D and DIC^4^D detector. Fig. 3A are the electropherogram of the K^+^ and Li^+^ solution of 2.5 μM (A_1_) and 250 mΜ (A_2_), respectively. Operating conditions: injected at 12 cm height for 5 seconds; separated at 10 kV in 20 mM MES-15 mM His buffer solution (pH 5.0); 200 kHz coupling frequency; 20 V (V_pp_) input AC voltage. Fig. 3B is the linear relationships of K^+^(B_1_) and Li^+^(B_2_) obtained by C^4^D and DIC^4^D with the range of 2.0 μM ~ 2.5 mM.

The C^4^D and DIC^4^D capillary electrophoretograms are shown in Fig. 3A. Better S/N and stable baselines are shown in both real cases at concentrations of 2.5 μM (Fig. 3A_1_) and 250 μM (Fig. 3A_2_). An obvious difference with C^4^D is that there are two group of peaks in the electrophoretograms of DIC^4^D, which were detected by two separated electrode pairs. The second group peaks appeared in time in agreement with C^4^D. The first group peaks appeared soon after the injection and had a narrow peak shape. According to the differences in shape, width, *et al*. of the two group peaks, the improved DIC^4^D can be used to detect the zone dispersion of analytes.

Figure 3B shows linear relationships of the K^+^ (B_1_) and Li^+^ (B_2_) obtained using C^4^D and DIC^4^D at different ion concentrations. DIC^4^D provides a better linear relationship (R = 0.997 and 0.998 for K^+^ and Li^+^, respectively) than C^4^D (R = 0.976 and 0.971 for K^+^ and Li^+^, respectively) with the range of 2.0 μM ~ 2.5 mM.

The baseline, noise and sensitivity of C^4^D and DIC^4^D in the above experiments were calculated and are listed in Table 1. Under identical experimental conditions, at the S/N = 3, the LODs of DIC^4^D are 1.0 μM for K^+^ and 1.5 μM for Li^+^. For C^4^D, the LODs are 1.8 μM for K^+^ and 2.5 μM for Li^+^. Because of factors such as different electrode processes and post-processing circuits, the LODs in our experiment may not be less than the results in some studies of C^4^D, but under the identical experiment conditions, compared to C^4^D, the improved DIC^4^D remains advantageous in terms of LODs. With the improvement of the electronic circuit, which includes the electrode and the amplitude of AC source, filter, *et al*., the method of DIC^4^D can decrease the LODs further more.

**Table 1:** Baseline, noise, sensitivity of C^4^D and DIC^4^D.

Detector	Baseline (mV)	Noise (mV)	Sensitivity (mV/μM)
C^4^D	102.051	0.005	1.4 × 10^−3^ (K^+^), 1.5 × 10^−3^ (Li^+^)
DIC^4^D	0.095	0.002	2.2 × 10^−3^ (K^+^), 2.3 × 10^−3^ (Li^+^)

The simulation and experimental results show that DIC^4^D performs better than C^4^D. The defect of the original DIC^4^D is overcome, and the improved DIC^4^D is validated for the application of capillary electrophoresis in this work. Although the actual DIC^4^D signal magnitudes in the experiment are not as large as the simulation, DIC^4^D performs better than C^4^D in the experiment, as expected from the simulation. The difference between experiment and simulation is the real value of physical parameters in the experiment, which is difficult to precisely obtain and easy to vary. In addition to depending on factors such as the structure of the impedance cell, analyte samples and carrier electrolyte solution, the physical parameters can be affected by the contact surface of the electrodes, geometrical shape, connection between cables and electrodes, and components in the detection circuit. The simulation only included some typical values for the C^4^D running parameters. However, the trends of change related to parameters are consistent with the experiment.

The theoretical consideration in this work explains the advantage of DIC^4^D. In the double-input circuit, the extra branch, which contains the AC voltage source and impedance, forms a parallel connection, which can decrease the outside impedance with respect to one AC source. Because the phases of the two sources are inversed, the parallel connection does not increase the current of the detection load, instead making the current almost zero. However, when the balance of the two inversed currents is broken, a small change in one branch impedance can cause a relatively large change in the detection load current. This change is also sensitive to the phase of the complex impedance of one branch. Thus, the measured signal using DIC^4^D includes the magnitude and phase change of the branch impedance. In contrast, C^4^D can only measure the magnitude change relative to the baseline.

Figure 3 shows the high S/N of DIC^4^D, which can be explained as follows. As mentioned in the reference^15,27^, the noise in C^4^D capillary electrophoresis mainly includes thermal noise, chemical noise from the chemical transformations during the electrophoretic run, noise from the signal generator, and ripple of the high-voltage source. The first two noise sources are considered common mode noise because they simultaneously occur for two electrode pairs, which can cancel each other. For C^4^D, there is an obvious low-frequency fluctuation or baseline drift because of these noises, whereas for DIC^4^D, the baseline drift is too low to be observed. In particular, the thermal noise and chemical noise, which are the main factors that cause the baseline drift^15,27^, can be removed by DIC^4^D. This advantage can be stably observed in our experiments. For the noise from the signal generator, because the two signal channels are relatively independent, some common electronic noise can be canceled, but independent noise remains in the two channels. For the last type of noise, the phase inversing does not act on the ripple from the driving high voltage source, so a low ripple voltage source is necessary here. Combining good electromagnetic shielding and a low ripple driving voltage source, DIC^4^D can obtain a better S/N.

Another obvious advantage of DIC^4^D is the high P/N. For DIC^4^D, because the basic coupled AC have been offset because of the inversed phases, the baseline is notably low. The low baseline favors the sequential amplifying and rectifying circuit. The amplifying circuit commonly has a limited linear range. The input signal beyond the range can cause saturation, which suppresses the small change overlapping a large base signal. This situation easily occurs for C^4^D, which weakens the effective signal and is also a main reason for the higher S/N and linear relationship of DIC^4^D compared to C^4^D.

### Concluding remarks

This work provides a theoretical explanation for the advantages of DIC^4^D, confirms the theoretical analysis with simulation, and validates the DIC^4^D detection method in the application of capillary electrophoresis. The improvement on the structure of DIC^4^D corrects the distortion in the peak shape, which is caused by two successive electrode pairs. The simulation and experiment also show the advantages of DIC^4^D, including the lower baseline, higher sensitivity, lower noise and good linear relationship compared to the generic C^4^D. Furthermore, according to the difference in peak shape and width between the two group peaks in the electrophoretograms, the improved DIC^4^D is expected to have the capability to measure the zone dispersion effects. The simulation and experimental results show the common mode noise suppression ability and dynamic range of the DIC^4^D structure C^4^D are better than those of the same coupling joint and amplifier acquisition circuit. It is believed that the lower detection limit of DIC^4^D can be achieved by improving the coupling efficiency. With the improvement of the electronic circuit, including the amplifier, filter, *et al*., DIC^4^D can be an attractive alternative in C^4^D application.

## Methods

### Chemicals and materials

Potassium chloride, lithium chloride, histidine (His), and 2-(N-Morpholino) ethanesulfonic acid (MES) were purchased from Kelong Chemical Co., Ltd. (Chengdu China). All chemicals were analytical grade or above and used as received without further purification. All solutions were freshly prepared with triply distilled water. A standard stock solution (1 M) of KCl and LiCl was prepared by dissolving the analyte in water. Standard solutions of KCl and LiCl were prepared by diluting the stock solution in water to give a series of concentrations in the range of 0.2 μM to 2.5 mM. The background electrolyte solution was 20 mM MES-15 mM His (pH 5.0). The sample and MES/His electrolyte were prepared daily.

### Apparatus

**Figure 4:**
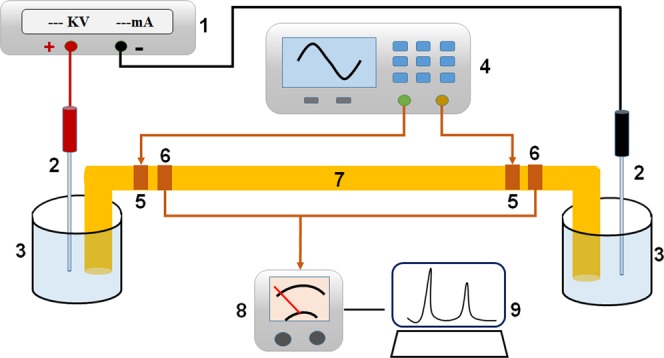
Schematic illustration of CE-DIC^4^D system. high-voltage power supply. 2. Pt electrodes. 3. buffer reservoirs. 4. function generator. 5. Input electrodes. 6. Output electrodes. 7. Separation capillary. 8. Millivoltmeter transformer. 9. computer.

Experiments were performed on an in-house made CE-DIC^4^D detection. The basic design is depicted schematically in Fig. 4. Briefly, high-voltage power supply (0–30 kV, Dong Wen High Voltage Power Supply Co., Tianjin, China) was used to drive the electrophoresis. A 80 cm × 75 μmI.D. × 365 μm O.D. uncoated fused-silica capillaries (Hebei Yongnian, Optical Fiber, Hebei, China) was used for the separation. Platinum wires serve as high-voltage electrodes. The Sinusoidal signal was produced two-channel function generator (DG4202, RIGOL Technologies, Inc.). The coupling signal from the electrodes was collected by a homemade Current/Voltage (I/V) converter, and was sent to a high-frequency millivoltmeter transformer (HFJ-8D, Shanghai Wuyi Electronics Co., Ltd.).

The structures of the impedance cell on the capillary are illustrated in Fig. 4. Two detection electrode pairs were fabricated by a copper wire wrapping pairs are located 15 cm from the two ends of the capillary tube. The contacted length of the electrodes was 4 mm, and the gaps between the two input electrodes and the output electrodes were 2 mm. To decrease the contact resistance and capacity, the gaps between the copper wires were filled with conductive paints. The electrodes and their locations in DIC^4^D were identical to those in C^4^D. To prevent the signal from environmental interferences, a strict electromagnetic shielding was used. Two ends of the capillary tube remained outside the shielding box to conveniently inject samples.

### CE procedure

The new capillary was rinsed with 1.0 M NaOH for 30 min before first use. Prior to the use each day, the capillary was successively preconditioned with 0.1 M NaOH for 10 min, water for 5 min, and background electrolyte for 10 min. The capillary was flushed with 0.1 M NaOH for 3 min, water for 2 min, and background electrolyte for 3 min between two consecutive injections. The sample solutions were injected into the capillary by hydrodynamic flow at a height of 12 cm for 5 s. Separations were performed at a constant voltage of 10 kV with 20 mM MES-15 mM His electrolyte solution (pH5.0), and the electric current is about 3 μA.

The coupling efficiency was measured using a pair of electrodes on a capillary tube filled with the buffer. AC sinusoidal wave signals of different frequencies were input, and the output signals were measured using an oscilloscope. The results show that the signal of 200 kHz had the best coupling rate. Thus, the input signals were 200 kHz, which were generated from a two-channel function generator. Two sinusoidals were imposed on both input electrodes. Their amplitudes were set to 20 V (Vpp), and the phases were set to 0° and 180°. In practice, the amplitude and phase must be adjusted in a small range to make the output alternative current signal zero on an oscilloscope, which was used as the monitor. The coupling signal from the electrodes was collected by a homemade I/V converter (Fig. 2A), sent to a high-frequency millivoltmeter transformer, and recorded by a data acquisition system. The driving current was stabilized at 30 mA.

### Simulation configuration

In this study, we performed a simulation implemented by using the MultiSim Circuit simulation tool (NI Company, Version No.10). We simulated the conductivity detection based on the entire circuit model, which included the AC signal source, equivalent circuit of C^4^D/DIC^4^D, and detection circuit.

Figure 2A_1_,A_2_ are the design circuits of C^4^D and DIC^4^D in MultiSim, respectively. In Fig. 2A, resistor R1 is the resistance in the capillary tube between two electrodes, capacitors C1 is the coupling capacitor, and C2 is the stray capacitor. The detection circuit included an I/V converter and the rectifying stage. The former consisted of an operational amplifier OPA2132P and a 1 MΩ feedback resistor R2; the latter is a Multimeter XMM1 with the rectifying function. Identical components are applied in Fig. 2A_2_ for DIC^4^D except for an extra AC source V2, whose initial phase has a 180° difference to that of V1, and an extra detection branch that consists of C3, C4, and R3.

The reactance parameters of the equivalent circuit of C^4^D/DIC^4^D were set up to be a series of typical values in experiments according to reference^3^. Here, we selected 1 MΩ R1 as the baseline resistance when there was only background electrolyte in the capillary tube. The capacitors C1 and C2 are 200 fF, 40 fF, respectively. For C^4^D, the detected effective signal is the coupled AC magnitude change caused by the analytes that move between two electrodes, which is represented by the change of R1. For DIC^4^D, R3 in Fig. 2A_2_ was assigned 1 MΩ to be the reference as a coinciding standard. The varying conductivity caused by the analytes is also represented by the change of R1 in Fig. 2A_2_. When the analyte flows through two electrodes, the conductivity correspondingly changes, and it hypothetically increases here. Thus, R1 of 0.9901 ~ 0.9259 MΩ was selected to investigate the linear response range of 1 ~ 10% conductivity increase. The signal of DIC^4^D is the voltage value measured by a multimeter XMM1. The signal of C^4^D is the change in coupled AC magnitude over the baseline and the voltage at other resistance values (0.9901 ~ 0.9259 MΩ) minus the voltage at 1 MΩ. To sufficiently investigate the frequency response, the input frequency was simulated from 100 kHz to 800 kHz at a 10% conductivity increase. Because, in our experiment, 200 kHz was the best coupling frequency (the largest output signal when the same magnitude AC signal is input), the signal response at different conductivities for different analyte concentrations was simulated at 200 kHz.

## Supplementary information

Supplementary Information.
