# Supplementary material for: Simulation and Experimental Study on Doubled-Input Capacitively Coupled Contactless Conductivity Detection of Capillary Electrophoresis
Source: Sci Rep. 2020 May 14;10:7944. doi: 10.1038/s41598-020-64896-3 (PMC7224287; doi:10.1038/s41598-020-64896-3)
Supplement: Supplementary file 1 — Supplementary Information. [file 41598_2020_64896_MOESM1_ESM.pdf]

Supporting Information

Simulation and Experimental Study on Doubled-Input Capacitively Coupled Contactless Conductivity Detection of Capillary Electrophoresis

Chunling Wang,[1] Haoyang Xing[2], Baozhan Zheng[3], Hongyan Yuan[1*], Dan Xiao[1,3*].

[1] College of Chemical Engineering, Sichuan University, Chengdu 610064, People's Republic of China.

[2] College of Physical Science and Technology, Sichuan University, Chengdu 610065, People's Republic of China.

[3] College of Chemistry, Sichuan University, Chengdu 610065, People's Republic of China.

This document provides extra information including:

Supporting information A: The detail of derivation of formulas in theoretical section.
Supporting information B: The detailed demonstration of the simulation circuit.
Supporting information C: The detailed procedure of double AC inputs adjustment in DIC^{4}D.

Supporting information A: The detail of derivation of formulas in theoretical section.

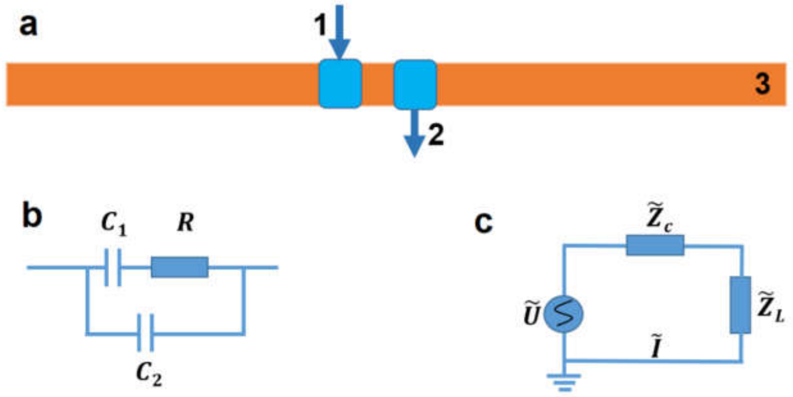

Figure S1. Schematic illustration of the structure, equivalent circuit and entire circuit model of a generic DIC[4]D.

The signal ($\tilde{I}_3\tilde{Z}_L$) can be resolved using Kirchhoff's Rule. According to Kirchhoff's Junction Rule, $\tilde{I}_3$ is the sum of $\tilde{I}_1$ and $\tilde{I}_2$, and the potential drops along the closed loops $\tilde{U}_1\tilde{Z}_1\tilde{Z}_L$ and $\tilde{U}_2\tilde{Z}_2\tilde{Z}_L$ are 0. Thus, three equations are expressed as:

$$\tilde{I}_1 + \tilde{I}_2 = \tilde{I}_3$$

$$\tilde{U}_1 - \tilde{I}_1\tilde{Z}_1 - \tilde{I}_3\tilde{Z}_L = 0$$

$$\tilde{U}_2 - \tilde{I}_2\tilde{Z}_2 - \tilde{I}_3\tilde{Z}_L = 0$$

The solution of $\tilde{I}_3\tilde{Z}_L$ is

$$\tilde{S}_{DIC4D} = \tilde{I}_3\tilde{Z}_L = \frac{\frac{\tilde{U}_1}{\tilde{Z}_1} + \frac{\tilde{U}_2}{\tilde{Z}_2}}{\frac{1}{\tilde{Z}_1} + \frac{1}{\tilde{Z}_2} + \frac{1}{\tilde{Z}_L}} = \frac{(\frac{\tilde{U}_1}{\tilde{Z}_1} + \frac{\tilde{U}_2}{\tilde{Z}_2})(\tilde{Z}_1\tilde{Z}_2\tilde{Z}_L)}{\tilde{Z}_1\tilde{Z}_2 + \tilde{Z}_1\tilde{Z}_L + \tilde{Z}_2\tilde{Z}_L} = \frac{\tilde{U}_1\tilde{Z}_2\tilde{Z}_L - \tilde{U}_2\tilde{Z}_1\tilde{Z}_L}{\tilde{Z}_1\tilde{Z}_2 + \tilde{Z}_1\tilde{Z}_L + \tilde{Z}_2\tilde{Z}_L} \tag{6}$$

The amplitude is

$$S_{DIC4D} = |\tilde{I}_3\tilde{Z}_L| = \frac{|\tilde{U}_1\tilde{Z}_2\tilde{Z}_L - \tilde{U}_2\tilde{Z}_1\tilde{Z}_L|}{|\tilde{Z}_1\tilde{Z}_2 + \tilde{Z}_1\tilde{Z}_L + \tilde{Z}_2\tilde{Z}_L|} \tag{7}$$

Selecting two identical-magnitude AC sources with inversed phases and using U to represent their amplitude, the signal amplitude can be written as

$$S_{DIC4D} = \frac{UZ_L|\tilde{Z}_2 e^{j(-\varphi_U)} + \tilde{Z}_1 e^{j(\pi-\varphi_U)}|}{|\tilde{Z}_1\tilde{Z}_2 + \tilde{Z}_1\tilde{Z}_L + \tilde{Z}_2\tilde{Z}_L|} = \frac{UZ_L|\tilde{Z}_2 - \tilde{Z}_1|}{|\tilde{Z}_1\tilde{Z}_2 + \tilde{Z}_1\tilde{Z}_L + \tilde{Z}_2\tilde{Z}_L|} \tag{8}$$

Supporting information B: The detailed demonstration of the simulation circuit.

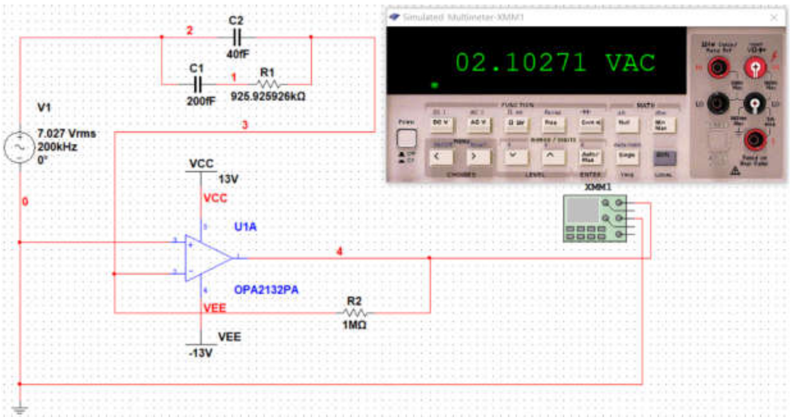

Figure S2-A. The circuit of C^{4}D simulation

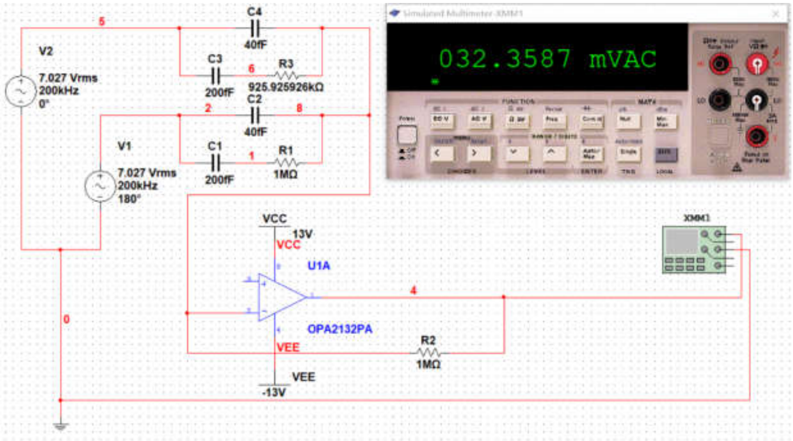

Figure S2-B. The circuit of DIC^{4}D simulation.

Figure S2-A and S2-B are the circuits with the parameters of components in Multisim 10.0 (National Instruments Corporate, Texas, USA). Here, an Agilent multimeter not ordinary multimeter in Figure 2 in the manuscript is adopted because the Agilent multimeter has 8-digit resolution vs 4-digit resolution of

default multimeter in Multisim 10.0. The ordinary multimeters were adopted in the manuscript to demonstrate the circuit principle. The format of AC voltage adopts rms (root mean square), and the corresponding peak-peak value is 20V. It is noteworthy that although the value on Figure S2-A is large to 2.10271 V, the effective value of C^4D is only value after minus the baseline 2.09376 V where R1 is 1MΩ.

Supporting information C: the detailed procedure of double AC inputs adjustment in DIC^4D.

In the experiment of DIC^4D, before injecting the sample, the two AC input channel of the signal generator are needed to be adjust to get the lowest baseline. On the panel of the signal generator, the frequency and amplitude of AC signals were set to be the value in the experimental section in the manuscript. Then the phase of one channel was set to 180º. However, because of the difference among the stray capacities of cables and lab made electrodes, the coupled amplitude and phase would be different to each other, the phase-inversed two signals cannot offset completely. So the amplitude and phase of two channels needed to adjust iteratively until very small magnitude just like the state of screen freezing picture shown in Figure S3-A. The coupled AC signal of C^4D was also shown in Figure S3-B as the control.

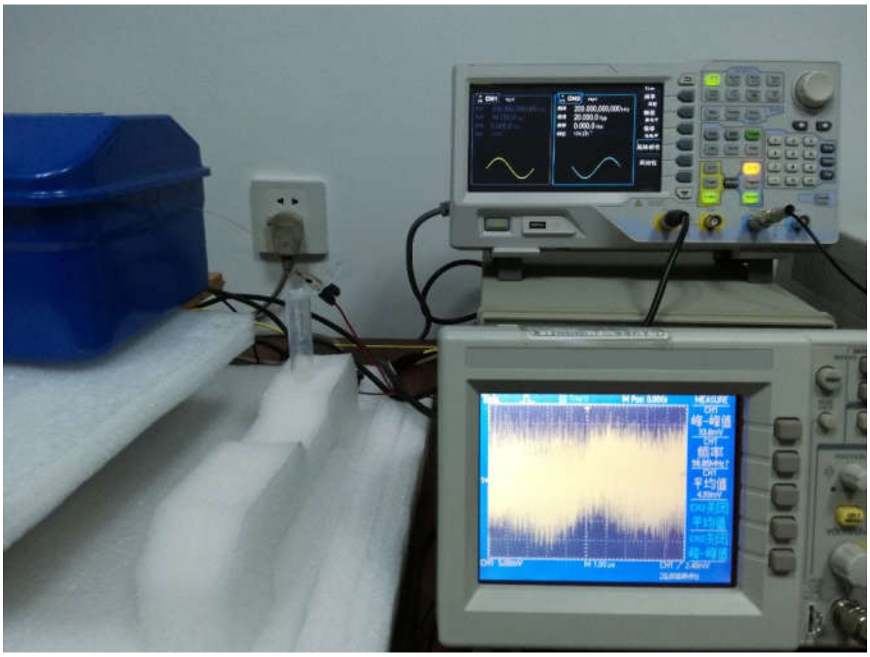

Figure S3-A. The coupled AC signal DIC^4D on the screen of oscilloscope.

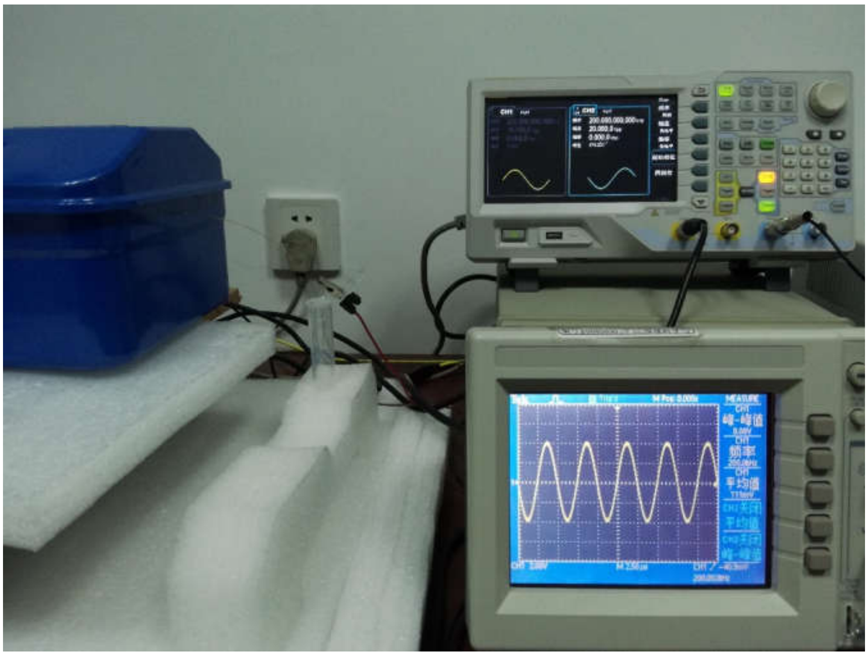

Figure S3-B. The coupled AC signal C[4]D on the screen of oscilloscope.
